# Insights on the Side Effects of Female Contraceptive Products From Online Drug Reviews: Natural Language Processing–Based Content Analysis

**DOI:** 10.2196/68809

**Published:** 2025-04-03

**Authors:** Nicole Groene, Audrey Nickel, Amanda E Rohn

**Affiliations:** 1 Department for Health and Social Sciences FOM University of Applied Sciences for Economics and Management Essen Germany; 2 VHC Health Arlington, VA United States

**Keywords:** contraception, side effects, natural language processing, NLP, informed choices, online reviews, women, well-being

## Abstract

**Background:**

Most online and social media discussions about birth control methods for women center on side effects, highlighting a demand for shared experiences with these products. Online user reviews and ratings of birth control products offer a largely untapped supplementary resource that could assist women and their partners in making informed contraception choices.

**Objective:**

This study sought to analyze women’s online ratings and reviews of various birth control methods, focusing on side effects linked to low product ratings.

**Methods:**

Using natural language processing (NLP) for topic modeling and descriptive statistics, this study analyzes 19,506 unique reviews of female contraceptive products posted on the website Drugs.com.

**Results:**

Ratings vary widely across contraception types. Hormonal contraceptives with high systemic absorption, such as progestin-only pills and extended-cycle pills, received more unfavorable reviews than other methods and women frequently described menstrual irregularities, continuous bleeding, and weight gain associated with their administration. Intrauterine devices were generally rated more positively, although about 1 in 10 users reported severe cramps and pain, which were linked to very poor ratings.

**Conclusions:**

While exploratory, this study highlights the potential of NLP in analyzing extensive online reviews to reveal insights into women’s experiences with contraceptives and the impact of side effects on their overall well-being. In addition to results from clinical studies, NLP-derived insights from online reviews can provide complementary information for women and health care providers, despite possible biases in online reviews. The findings suggest a need for further research to validate links between specific side effects, contraceptive methods, and women’s overall well-being.

## Introduction

### Background

According to the United Nations, contraception is a critical issue impacting 1.9 billion women of reproductive age. Worldwide, approximately 922 million women or their partners use contraception. More than half of all contracepting women rely on modern contraceptive products designed to be used by women. These female products comprise long-acting reversible contraceptives (LARCs), such as intrauterine devices (IUDs) and hormonal implants as well as short-acting methods, such as oral contraceptives, known as “the pill,” hormonal patches, vaginal rings, and contraceptive injections. Traditional methods such as withdrawal and calendar rhythm are relied upon by 7% of women, and the single most common contraceptive method worldwide is female sterilization (24%), an irreversible method [1,2].

According to data from the latest National Survey of Family Growth (2017 to 2019), approximately 27.5% of women of reproductive age in the United States use female contraceptive products, comprising oral contraceptive pills (OCPs, 14%), LARCs (10.4%) and other short-acting methods, such as contraceptive injections (2%), vaginal rings (0.8%), and patches (0.3%) [2]. With increasing levels of formal education, the prevalence of LARC and short-acting methods increases while the prevalence of female sterilization decreases [3].

While female contraceptive products are reversible and generally more efficacious than traditional methods, thus offering advantages to women with regards to their family planning and thus self-determination [1,4], they can be associated with unpleasant experiences [5,6], ranging from abdominal pain to mood swings or changes in libido [7,8]. The experience of such unpleasant side effects has a negative impact on a woman’s health, which the World Health Organization defines as “state of complete physical, mental, and social well-being,” and thus on the quality of life [9,10]. Furthermore, negative side effects are a major cause for poor adherence or even discontinuing contraception which may result in unintended pregnancies [11,12].

### Access to Data to Inform Contraceptive Choices

For women to find the contraceptive method that is most suitable for them and thus make informed contraception choices, it is important to have access to relevant information regarding different available contraception options. The type of information that women require can be assigned to 2 broad categories.

First, information relating to the efficacy of contraceptive methods regarding preventing unintended pregnancies and protection from sexually transmittable diseases is crucial [13]. There is comprehensive clinical as well as real-world data on efficacy and safety of different contraceptive methods [14,15]. These data are generally accessible to women through health care providers (HCPs) or nongovernmental organizations, although there are geographical differences on a global level [16].

Second, women seek information relating to potential unpleasant experiences related to contraceptive methods as these can have a substantial negative impact on women’s well-being, not only impacting women themselves, but also their families [13,17,18]. However, there are 2 major challenges women face when seeking information about potential negative experiences related to contraceptive methods, namely, the availability of data and the accessibility of reliable data [17].

Data on the frequency of negative side effects are generally available, as they are collected in clinical trials and stated on drug labels [19,20]. However, the construct of well-being is more nuanced, comprising a “state of positive feelings and meeting full potential in the world” [21]. Consequently, data on the mere occurrence and frequency of certain side effects provide insufficient information on how certain side effects typically impact well-being. For example, abdominal pain related to a contraceptive product might constitute a neglectable nuisance or a major suffering limiting women’s participation in daily life. Despite the subjective nature of side effect severity [22], for women facing contraception choices, knowing that a certain side effect can be a significant issue for some women constitutes relevant information [17]. However, there is a lack of comprehensive data on women’s subjective and collective unpleasant experiences with different contraceptive methods [23]. Studies have also shown that while women tend to turn to HCPs for contraceptive counseling, HCPs often lack relevant knowledge and provide insufficient information on potential side effects [24,25]. To learn about experiences with different contraceptive methods, women also tend to speak to relatives and friends, but these experiences are subjective and constitute a small sample size.

### Role of Social Media to Inform Contraception Choices

From this background, social media has started to play an important role as a source of information. Experimental research indicates that social media content may influence women’s intentions to use certain contraceptive products [26] even as social media conversations about contraception have become more polarized in the past 20 years [27].

Thus, there is a growing body of research to evaluate how women use social media to inform contraception choices [28]. To analyze information shared and consumed on social media, natural language processing (NLP) is used due to its capacity to analyze nonstructured, textual data.

For example, Pleasants et al [28] used NLP to study posts related to birth control on the US platform Reddit and found that “Side Effects!?” is the most common flair, a tag that users can attach to their post to categorize the content. Furthermore, “Experience” and “Side Effects?!” are the most common flairs among the most popular posts, based on the number of comments and “scores,” that is, upvotes minus downvotes [28]. Analyzing contraceptive content shared on X (formerly Twitter) Huang et al [29] discovered that a fifth of all the tweets relate to side effects. Similarly, in a text mining analysis of messages sent on a free sexual and reproductive health information service in Kenya, Green et al [30] found that users wrote most often about family planning and side effects. Balakrishnan et al [31] conducted an NLP-based social listening analysis in a German internet community and observed that side effects are the most common problem associated with most contraceptives. They also found that while the pill is the most frequently mentioned contraceptive method, there appears to be migration from hormonal to nonhormonal methods. In line with this, Felice et al [32] analyzed user reviews of a digital contraceptive supporting women in fertility prediction through a mixed methods approach involving NLP and found that the hormone-free aspect of the contraception experience is highly salient for many users. A content analysis by Pfender and Devlin [33] of YouTube vlogs discussing birth control methods revealed that social media influencers primarily described their discontinuation of hormonal birth control due to experienced side effects. Their study also showed that vlogs may provide inaccurate sexual health information, hereby directly or indirectly discouraging the use of the contraceptive under discussion. In a content analysis of the “sex secrets” Facebook page, Yeo and Chu [34] found that young people predominantly use this social media platform to request information, opinion, or advice, including the topic of birth control. Stoddard et al [35] found that more than half of the most popular contraception videos on TikTok revolved around patient experience. Although videos created by health care professionals received proportionately more views, over half of the total views were still of content generated by laypeople [35].

Overall, these NLP-based social media content studies show that social media is used to share information and consume information on contraception options. Furthermore, they reveal that user-generated content mostly revolves around side effects and that posts discussing women’s experiences with regards to side effects receive the greatest interest. At the same time, the content that is available, especially when shared by influencers, is not always reliable and may misguide contraception choices. In fact, there is increasing concern among researchers and women’s health practitioners that social media influencers spread misconceptions about contraceptive methods, particularly hormonal contraception, which negatively affect the acceptance of efficacious contraceptive methods and thus increase the risk of unintended pregnancies [36].

Furthermore, the previously mentioned studies highlight that unpleasant experiences are an issue that is currently not well-addressed in clinical contraceptive counseling. This further substantiates the observation that users appear to have an unmet need for reliable, trustworthy information. However, existing NLP-based studies do not provide a systematic picture of the association of different side effects with different available contraceptive methods and how severely women experience these side effects.

To fill this gap, the NLP method of sentiment analysis can identify, extract, and quantify the subjective emotions within a text, assigning a continuous sentiment score usually between −1 for highly negative and 1 for highly positive posts [37,38]. Studies using sentiment analysis may thus provide first hints as to how severely women experience certain side effects. Merz et al [27] studied population attitudes toward contraceptive methods over time by performing sentiment analysis on tweets on X regarding contraceptive methods and find that most tweets are negative. In their sample long-acting methods are mentioned more often than short-acting ones and related sentiments are twice as likely to be positive [27]. In contrast, in a study with Indonesian users of X, Sari et al [39] found that users predominantly express negative attitudes toward long-acting contraceptive methods.

However, a major limitation of sentiment analysis is that it can be inaccurate if the model has been trained on biased, limited, or unrepresentative datasets as it may fail to generalize well to diverse and nuanced language usage, such as sarcasm, slang, or cultural context variations present in social media posts. Although the modern state-of-the art approach in sentiment analysis involves using pretrained language models such as Bidirectional Encoder Representations from Transformers or GPT, there is an inherent risk of bias in general and gender bias in particular [40,41], limiting performance in sentiment analysis tasks.

In this context, the information on online drug review forums constitute a great, widely untapped, resource to inform contraception choices. Many online drug review forums contain 2 distinct pieces of information related to a product: a standardized numeric rating score indicating overall product satisfaction and a comment in free text form. A powerful advantage of online product reviews is that the integration of qualitative (text comments) and quantitative (ratings) data facilitates insights into the relationship between issues mentioned in comments and overall product satisfaction, which is presumably closely linked to the impact of the respective product on the well-being of the user.

Evaluating data from online review forums to inform decisions is hampered by several limitations, such as potential biases, unrepresentative sample issues, and the potential presence of inauthentic reviews. Nevertheless, consumer behavior in many industries, including health care and retail, indicates that other people’s reviews, particularly when available in large numbers, are important in driving purchase decisions and are thus considered a valuable source of information [42]. Thus, in the context of contraception, reviews data complement information on contraceptive options that women and their partners may receive from other sources, such as HCPs, community workers, scientific studies, or other social media sources.

### Purpose of the Study

This research aimed to produce insightful information from a large drug review dataset with regard to which experiences with contraceptive products women described on the web, both qualitatively and quantitatively. The focus is on unfavorable experiences, as previous research has shown that side effects are the topic of greatest interest for women using forums and social media to seek information on contraception.

From this background, in this paper, we investigated the following research questions (RQs):

1. How do users rate different contraceptive methods available to women on a major drug review website?
2. Which issues (ie, topics) do users describe in unfavorable online reviews of contraceptive products available to women?
3. How frequently are these issues described for different contraceptive methods?
4. Can we observe an association between the main topic discussed and the average rating submitted in unfavorable birth control reviews?

## Methods

### Dataset

Our study was performed on a dataset of 19,506 unique online reviews of birth control products in the United States posted on the website Drugs.com [43], a United States–based pharmaceutical information website, between April 2009 and September 2017. The reviews analyzed in this study were extracted from a comprehensive online drug review dataset available for research purposes in the University of California, Irvine (UCI) Machine Learning Repository [44]. The original dataset had been collected via web scraping from the website Drugs.com [43] and comprised 215,063 reviews of drugs treating different conditions, such as high blood pressure, cough, and birth control [45]. While this dataset may be somewhat dated, these reviews are highly relevant for this study. First, the products evaluated have been on the market for many years and are widely used today. Second, analyzing older reviews might even offer the advantage of capturing women’s experiences with contraceptive products in a more authentic, less skewed way. Research has shown that in recent years, social media influencers negatively frame hormonal contraceptives and encourage the uptake of nonhormonal options which may alter women’s attitudes and expectations [26] and thus possibly their online reviews.

The online drug user reviews contained information on the related condition, the name of the drug, a 10-star user rating on overall satisfaction, how many users considered this review helpful, and the date the review was posted. The name of the drug was captured in a structured format as it stemmed from a drop-down menu from which the website users needed to select a drug name when leaving a comment.

### Ethical Considerations

The study used publicly available data from the UCI Machine Learning repository. The UCI Machine Learning dataset did not contain any identifying information about the authors of the reviews, such as their username. Furthermore, when posting a review on the website Drugs.com [43], users were required to consent to the publication and use of their reviews. Finally, to the best of our knowledge, the reviews we selected to be in this manuscript do not risk reverse identification as the website Drugs.com [43] does not display full user names alongside the reviews. Therefore, in line with other studies evaluating social media posts on contraception, ethics approval for using these reviews as a basis for analysis was not deemed necessary.

### Data Cleansing and Grouping

Within the drug review dataset offered by the UCI Machine Learning Directory, 38,436 product reviews were classified as relating to “birth control.” Many reviews were captured twice, once under a product’s brand name and a second time under the name of the respective active pharmaceutical ingredient, that is, the generic name. By removing duplicates, we obtained 19,524 unique birth control reviews. When cleansing the dataset, we retained drug brand names for their greater detail compared to generic names. This granularity is more suitable for analysis, as products with the same active ingredient can vary in dosage and administration schedules across brands. In total, <400 out of 19,524 unique reviews did not contain a brand name, but rather only the generic name. We kept most of those reviews in the dataset, only removing 13 unique reviews of drugs that could not be related to 1 specific contraceptive method, namely, levonorgestrel (10 reviews), which can be a hormonal IUD or emergency contraception commonly sold as Plan B; and Provera (3 reviews), which can either be a birth control shot under the name Depo-Provera or an oral progestin product that is not approved as a contraceptive. The clean birth control dataset contained unique reviews on 169 different products identified by brand name or active pharmaceutical ingredients.

For later analysis and comparison of drug reviews for different contraceptive methods, we categorized all products into 11 contraceptive methods. This categorization focused on the application mode of these products, which is in line with the classification of contraception options typically used for advising women [46]. The methods comprise: hormonal and nonhormonal (ie, copper) IUDs, implants, vaginal rings, birth control shots, hormonal patches, spermicides, and emergency contraception. For OCPs, we distinguished between combined contraceptive oral pills (COCPs), progestin-only pills (POPs), and OCPs that induce a 91-day cycle, as these are expected to have different side effect profiles, and patients are typically counseled differently. Given the small number of reviews on emergency contraception (n=3) and spermicides (n=2), we removed those reviews from the dataset, too, leaving 19,506 reviews on 167 different products across 9 different contraceptive methods.

To analyze which negative experiences or side effects related to birth control options women described, we created a new attribute marking all reviews with a rating of ≤5 (on a scale from 1 to 10) as unfavorable reviews. Rather than limiting our analysis to reviews associated with strictly negative ratings (usually defined as ≤3), we deliberately used a wider window to also include negative to neutral ratings (scores of 4 and 5) as these might also contain relevant descriptions of unpleasant experiences. Overall, 8330 reviews fell into our definition of unfavorable (ie, nonpositive with a rating of 5 and lower).

### NLP Approach for Analyzing Unfavorable Birth Control Reviews

#### Overview

Within NLP, topic modeling refers to techniques for uncovering abstract themes in a large textual dataset, typically referred to as a corpus. It involves algorithmically analyzing documents to detect word and phrase patterns that indicate specific topics. Thus, topic modeling allows analyzing which topics women discuss in unfavorable reviews of different contraceptive products. For our study, we wrote an NLP program for topic analysis in Python (version 3.11.3; Python Software Foundation) using several NLP libraries, including Natural Language Toolkit (version 3.7) [47] and scikit-learn (version 1.2.2) [48]. For visualization, we used Matplotlib (version 3.7.1) [49] and Seaborn (version 0.12.2) [50].

#### Text Preparation

Our text preprocessing procedure included multiple steps. Text cleaning was performed as the raw birth control reviews in the UCI repository contained several issues with punctuation and how certain characters were captured. Furthermore, we implemented a custom-developed catalog of more than 110 abbreviations and short forms to replace them with the long form. Examples include “can’t” being replaced with “cannot,” “PMS” with “premenstrual syndrome,” “yr” with “year” or “ain’t” with “am not.” If an abbreviation had 2 meanings, such as “he’s,” we replaced it with the most common form. This step ensured uniformity in word representation so that the frequency of a word could be captured adequately. In addition, we removed any nontext characters, created word tokens and reduced words to their base root via lemmatization. To further reduce the dimensionality of the textual data and focus on the most meaningful words, we excluded common words typically not carrying meaning, so-called “stop words” as predefined in NLTK, except for the stop word “not” which adds to the meaning of a review describing potential complaints or side effects. We also removed all product names and contraceptive methods, such as “iud,” “implant” or “pill,” from the reviews to allow our topic modeling algorithm to reveal topics that are contraceptive product and method agnostic.

As Table 1 shows, after the removal of stop words, there were highly frequent words in the reviews that did not relate to specific birth control side effects or complaints. To reduce noise and dimensionality, we removed the words “month,” “day,” “year,” “week” “birth,” and “control” from the reviews.

**Table 1:** Most common words in the birth control product reviews (excluding noninformative words).

Word	Occurrences (n=889,864), n (%)	
not	30,973 (3.48)	
period	19,145 (2.15)	
month	18,361 (2.06)	
day	11,139 (1.25)	
control	10,902 (1.23)	
birth	10,678 (1.2)	
year	9986 (1.12)	
week	9464 (1.06)	
first	8702 (0.98)	
get	8020 (0.9)	
weight	7783 (0.87)	
would	7146 (0.8)	
got	7061 (0.79)	
time	6956 (0.78)	
like	6410 (0.72)	
side	6186 (0.7)	
effect	5982 (0.67)	
cramp	5704 (0.64)	
started	5545 (0.62)	
since	5332 (0.6)	
mood	5302 (0.6)	
taking	5292 (0.59)	
bleeding	5278 (0.59)	
acne	5156 (0.58)	
never	5018 (0.56)	

#### Topic Extraction Approach

In topic modeling, selecting the optimal vectorization techniques, topic modeling algorithms, and the number of topics to extract is crucial. This process aimed to derive topics that align with domain-specific inquiries and RQs. While coherence and silhouette scores can support this selection, domain expertise and expert judgment are essential in evaluating the relevance and applicability of the themes extracted by an algorithm [51].

The topics described in the following sections result from an iterative strategy combining various vectorization techniques to construct a document-term matrix, including count vectorization and term-frequency–inverse document frequency (TF-IDF). We used topic modeling algorithms, such as latent semantic analysis, nonnegative matrix factorization (NMF), and latent Dirichlet allocation, extracting between 3 and 13 topics. The selection of techniques and the number of topics was based on expert judgment, Cohen coherence, and the silhouette score, with the final decision guided by domain knowledge to yield the most useful, interpretable, and distinct topics.

The final technical configuration of the topic modeling in this research is as follows:

- Vectorization—TF-IDF vectorization yielding a document-term matrix, where rows represented reviews, columns represented words, and values indicated word importance. TF-IDF highlights terms frequent in a document (ie, a product review) but less common across the corpus (ie, across all reviews), reducing the weight of ubiquitous words.
- Topic modeling algorithm—NMF decomposing the nonnegative document-term matrix into 2 lower-dimensional matrices: the topic matrix (W) and the terms matrix (H). The topic matrix represented documents by underlying topics, while the terms matrix represented topics by original words or tokens.
- Number of topics—8 topics differentiating most effectively among various types of experiences and complaints.

**Figure 1:**
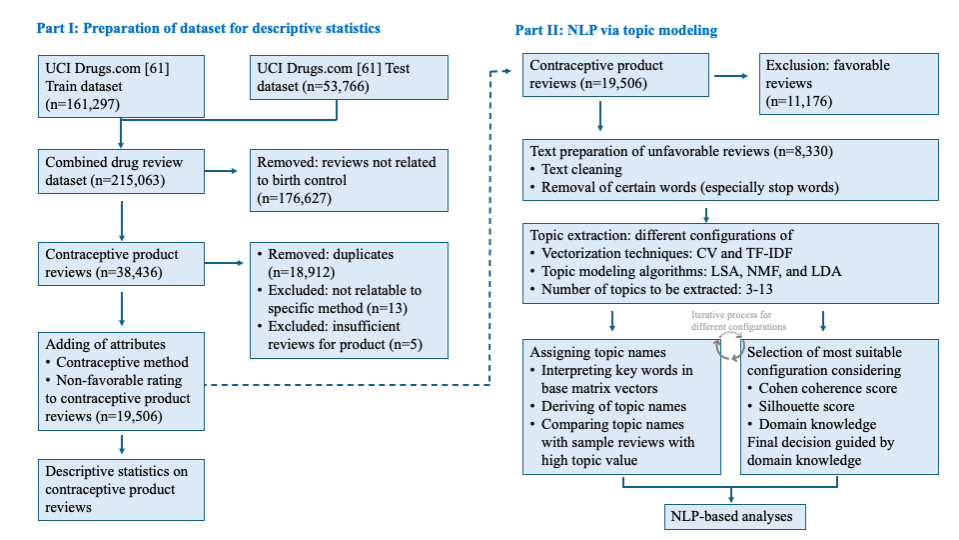
A flow diagram of the methodological approach used in the study. CV: count vectorization; LDA: latent Dirichlet allocation; LSA: latent semantic analysis; NLP: natural language processing; NMF: nonnegative matrix factorization; TF-IDF: term-frequency–inverse document frequency; UCI: University of California, Irvine.

The flowchart in Figure 1 illustrates the overall methodological approach.

## Results

Descriptive and topic analysis of the website Drugs.com [43] drug reviews dataset allowed us to answer our RQs.

### Online Ratings of Different Contraceptive Methods Available to Women (RQ 1)

#### Frequency of Ratings

Table 2 displays the distribution of ratings of birth control products in the drug review dataset from 1 to 10, with 1 being very bad and 10 being very good. The frequency diagram of the ratings is U-shaped, such that both very poor and very good ratings were common. The most common rating was 10 out of 10 (n=3905, 20.02% of the reviews), and the second most common rating was 1 out of 10 (n=2986, 15.31% of the reviews). The overall mean was 6.08, and the SD was 3.31. Thus, reviews were polarized, but on average gravitated toward positive ratings.

**Table 2:** Frequency of contraceptive product ratings on a scale from 1 to 10 (1: very bad and 10: very good) in the online drug review dataset (n=19,506).

Rating	Frequency, n (%)
1	2986 (15.31)
2	1409 (7.22)
3	1363 (6.99)
4	1083 (5.55)
5	1489 (7.63)
6	964 (4.94)
7	1253 (6.42)
8	2112 (10.83)
9	2942 (15.08)
10	3905 (20.02)

#### Ratings of Different Contraceptive Methods

Table 3 provides an overview of the number of available birth control product reviews in the dataset, grouped by the product categories. COCPs are the most reviewed birth control products, constituting 44.12% (8606/19,506) of all reviews. Hormonal implants and hormonal IUDs rank second and third, respectively.

Slightly more than half of birth control product reviews (n=11,176, 57.3%) are favorable according to our definition, whereas 42.7% (8330) of reviews are unfavorable. Overall, the share of unfavorable reviews varied substantially across categories. POPs had the highest share of unfavorable reviews (n=232, 53.1%), whereas nonhormonal IUDs had the lowest (n=234, 29.3%).

**Figure 2:**
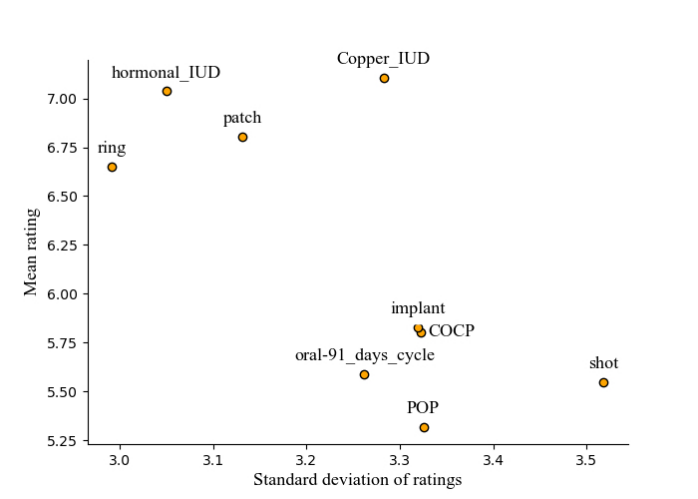
A scatter diagram with trimmed axes visualizing average rating and SD of different contraceptive methods. COCP: combined contraceptive oral pill; IUD: intrauterine device; POP: progestin-only pill.

Figure 2, a scatter diagram with trimmed axes, reveals 2 clusters of different contraceptive methods based on mean ratings and SDs. The first cluster, located in the lower right, includes POPs, birth control shots, 91-day cycle OCPs, COCPs, and hormonal implants, with lower average ratings (5.32-5.82) and higher SDs (3.26-3.52). The second group, situated in the upper left, comprises hormonal and copper IUDs, hormonal patches, and vaginal rings, exhibiting higher average ratings (6.65-7.11) and generally lower SDs (2.99-3.13), except for copper IUDs, which had a SD of 3.28.

**Table 3:** Descriptive statistics of product ratings by contraceptive method.

Contraceptive method	Unique reviews (n=19,506), n (%)	Products (n=167), n (%)	Rating, mean (SD)	Number and share of unfavorable reviews (n=8330, 42.7%), n (%)
COCP^a^	8606 (44.12)	138 (82.6)	5.80 (3.32)	3968 (46.11)
Implant	4392 (22.52)	3 (1.8)	5.82 (3.32)	2064 (46.99)
Hormonal IUD^b^	2871 (14.72)	4 (2.4)	7.04 (3.05)	848 (29.54)
Vaginal ring	827 (4.24)	2 (1.2)	6.7 (3.0)	297 (35.91)
Copper IUD	800 (4.1)	2 (1.2)	7.11 (3.3)	234 (29.25)
Shot	653 (3.35)	2 (1.2)	5.5 (3.5)	327 (50.08)
Patch	508 (2.6)	3 (1.8)	6.8 (3.1)	157 (30.91)
POP^c^	437 (2.24)	11 (6.6)	5.3 (3.3)	232 (53.09)
OCP^d^ with 91 d cycle	412 (2.11)	9 (5.4)	5.6 (3.3)	327 (49.27)

^a^COCP: combined contraceptive oral pill.

^b^IUD: intrauterine device.

^c^POP: progestin-only pill.

^d^OCP: oral contraceptive pill.

### Issues Described by Users in Unfavorable Online Reviews of Contraceptive Products Available to Women (RQ 2)

Table 4 presents the 8 themes extracted from the 8330 unfavorable birth control product reviews in our dataset. Overall, each extracted theme corresponded to a description of side effects. There was no topic that explicitly alluded to nonhealth aspects such as cost, ethical or societal concerns, or accessibility. A total of 4 topics extracted were highly specific and related to “weight gain,” skin problems,” “loss of libido,” and “mental health problems.” Another 3 topics related to the impact of the contraceptive product on women’s menstrual cycle but alluded to distinct aspects, which we named “menstrual irregularities,” “cramps and pain,” and “continuous bleeding.” The final topic, “multiple cause dissatisfaction,” was a mixed, broad topic. It was the least distinct topic, comprising a mixture of diffused complaints ranging from headache, tiredness, general life, and relationship issues to a mere product warning. A sample review that scored high on the topic “multiple cause dissatisfaction” read as follows:

Makes me feel very moody and sensitive, my husband and I fight all the time. When we got married I felt so much in love but know not sure about it. He said I changed a lot after having our baby. So not sure if the IUD is making me feel that way. I feel so bad because I get mad very easy for little things and I feel like I am loosing my husband. Of course that he doesn’t want to wear his ring makes me think things but he said that he is not use to wear rings and I always wear mine. I cook breakfast every single day, cook lunch for us to take it to work since we do not have to much money and sometimes I feel that he doesn’t really appreciate it! Do laundry, clean and he doesn’t really help me much and he doesn’t see it. Not sure what to think.

Table 5 provides sample reviews for each topic. More examples can be found in Multimedia Appendices 1 and 2.

For each topic, Table 4 also presents the share of online user reviews where this topic was dominant having the highest topic value in the topic matrix W. Thus, the dominant topic is the issue voiced most firmly in an unfavorable review. Table 4 shows that with this dominant topic modeling scheme, the reviews were relatively evenly distributed among the 8 identified topics. The rarest dominant topic was “weight gain” with 9.69% (807/8330) of the unfavorable reviews predominantly describing this side effect. “Multiple cause dissatisfaction” dominated in 17.92% (1493/8330) of the unfavorable reviews.

**Table 4:** Topics discussed in unfavorable reviews of birth control products and their relative frequency (n=8330).

Topic	Topic description	Unfavorable reviews with this dominant topic, n (%)
Weight gain	Users describe a change in body weight, typically an increase, which is attributed to the contraceptive product	807 (9.69)
Skin problems	Users describe an impact of the product on outward appearance, in particular acne	1051 (12.62)
Loss of libido	Users describe a reduction or loss of interest in physical intimacy and intercourse	963 (11.56)
Mental health problems	Users describe mental health problems, such as mood swings, depression, and anxiety	902 (10.83)
Menstrual irregularities	Users describe different problems with their period resulting from the contraceptive method; ranging from spotting, heavy bleeding, to unusually light periods	1223 (14.68)
Cramps and pain	Users describe particularly painful experiences, especially cramps, associated with the product or its administration	926 (11.12)
Continuous bleeding	Users describe continuous bleeding episodes which last substantially longer than regular periods and are more pronounced than simple menstrual irregularities	965 (11.58)
Multiple cause dissatisfaction	Users express dissatisfaction with the contraceptive product. None or various reasons are provided ranging from general side effects such as headaches to overall challenges in life that might or might not be attributable to the contraception choice	1493 (17.92)

**Table 5:** Examples of reviews centering on a specific topic.

Topic	Sample reviews with the dominant topic
Weight gain	“Been on it for 3 months, 20 pound weight gain—always hungry and never full. No periods, but not worth the weight gain and uncontrollable appetite...Was managing weight very well prior to implant...”
Skin problems	“Horrible, horrible, horrible I have never had acne this bad in my life!!!!!!!!!! My WHOLE chin and jawline are red and covered in cystic acne!!! I HAD PERFECTLY CLEAR SKIN BEFORE. I am honestly in a complete panic with what is going on with my skin. I’m in shock that a small pill could do this much damage. My face hurts so bad because of the acne. Its been only 3 weeks since I started taking it. Switching to sprintec tomorrow. DO NOT USE THIS, SAVE YOUR SKIN!!!!!”
Loss of libido	“I have been on NuvaRing for 5 months. Within a month I noticed a decrease in my sex drive, and I’ve had vaginal dryness which makes sex painful. Bad sex has effected other parts of my life.”
Mental health problems	“I used this pill during my teens and it caused irritability and heavy mood swings. Perhaps it was just teen angst but I tried microgynon recently, which uses the same hormones just different levels, and experienced similar mood swings and depression.”
Menstrual irregularities	“I have been on this medication for almost a month. I got my period once, but it hasn’t even been a week later that I got a second period. My first period was very light and only lasted three days, but I’m not sure how this period will be.”
Cramps and pain	“I got the kyleena inserted today and experienced the worst cramps in my life. The insertion were (8/10) on the pain scale. I am not very sensitive to pain but can’t take any pain medication. The last 4 hours has been the worst in my entire life so far I have really bad cramps now 10/10 and nausea. I can’t even get out of bed because of the severe pain!”
Continuous bleeding	“With liletta I have been bleeding for 3 month s I am so so tire of bleeding.”
Multiple cause dissatisfaction	“Do not take this pill.”

### Relative Frequency of Side Effects Described Across Contraceptive Methods (RQ 3)

**Figure 3:**
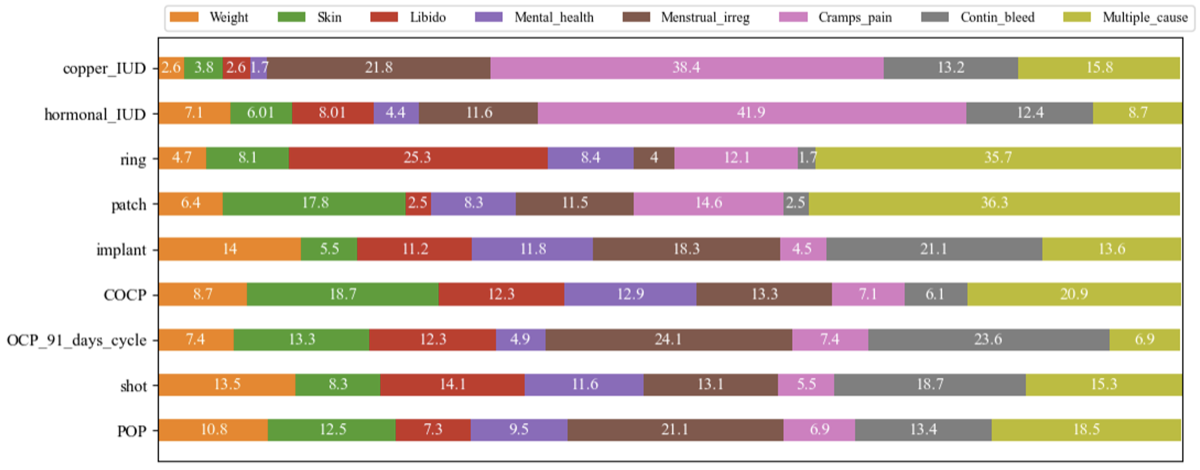
Relative frequency of dominant topics in nonfavorable reviews by contraceptive method (as percentages). COCP: combined contraceptive oral pill; IUD: intrauterine device; POP: progestin-only pill.

For each contraceptive method, Figure 3 shows the relative frequencies of the dominant topics in descending order according to average rating. For both copper and hormonal IUDs, the most frequent complaint by far was “cramps and pain,” which was the dominant theme in 38% (n=90) and 42% (n=355) of the reviews, respectively. For COCP, the most common complaints were “multiple cause dissatisfaction” (n=831, 21%) and “skin problems” (742, 19%). For POP and OCP that induce a 91-day cycle, “menstrual irregularities” were the most common issue (n=49, 21%, and n=49, 24% of reviews, respectively). For implants, as well as for hormonal shots, “continuous bleeding” (n=436, 21%, and n=61, 19%, respectively) was the most frequent problem described in the reviews. For hormonal patches and vaginal rings, the most frequent dominant topic was “multiple cause dissatisfaction” (n=57, 36% and 106, 36%). For hormonal patches, the second most frequently described dominant side effect prominently voiced in unfavorable online reviews was “skin problems” (n=28, 18%).

When reviewing the relative frequencies of dominant topics identified in Figure 3, it is important to remember that each contraceptive method was associated with a different proportion of unfavorable reviews. This was analyzed in the context of RQ 1 and is depicted in Table 3 which displays substantial variation in the proportion of unfavorable reviews across contraceptive product categories; with POP having the highest share of unfavorable reviews (n=232, 53.1%) and copper IUDs having the lowest share of unfavorable reviews (n=234, 29.3%). Scaling the relative frequencies of the dominant topics shown in Figure 3 with the overall share of unfavorable reviews of contraceptive methods displayed in Table 3, we find that for certain contraceptive methods, specific issues were very commonly discussed in online reviews in general, as in the following examples:

- Almost a quarter, that is, 24% (n=97), of all reviews of 91-day cycle OCPs report general “menstrual irregularities” or “continuous bleeding” (this is derived from n=49, 24%, of the reviews where “menstrual irregularities” were the dominant topic plus another n=48, 24%, where “continuous bleeding” was the dominant topic; multiplied by 49.3%, the rate of unfavorable reviews).
- Overall, 17% (n=104) of all reviews of hormonal shots discussed “menstrual irregularities” or “continuous bleeding.”
- For IUDs, 12% (n=90, copper) and 11% (n=355, hormonal) of all reviews revolved around “cramps and pain” associated with the contraceptive method and its administration.
- “Loss of libido” was the dominant topic in almost every 10th review of vaginal rings, that is, 9% (n=75).
- Almost 6% (n=243) of all reviews of “hormonal implants” revolved primarily around mental health issues.

### Association Between Dominant Topic and Ratings of Birth Control Products (RQ 4)

The final RQ relates to how severely such side effects might impact the well-being and overall quality of life of women. This approach is important for providing a balanced picture of the frequency numbers described earlier. Not every side effect, even if common, necessarily impacts overall well-being to the same extent. For example, individual reviews illustrate that “cramps and pain” might have a much more negative impact on overall well-being than menstrual irregularities. For illustration, a sample review with “cramps and pain” as the dominant topic read as follows:

My experience was absolutely horrible. Birth control works different for everyone but this was by far the worst pain I’ve ever been in...

While a sample review where menstrual irregularities were voiced read as follows:

I have been on this medication for almost a month. I got my period once, but it hasn’t even been a week later that I got a second period. My first period was very light and only lasted three days, but I’m not sure how this period will be.

**Figure 4:**
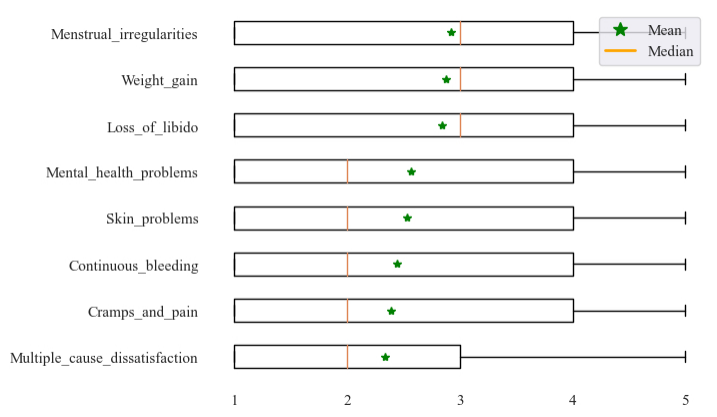
Boxplots of ratings by dominant topic described in nonfavorable reviews.

Figure 4 displays boxplots of unfavorable ratings by dominant topic. The boxplots show that the frequency distributions for all dominant topic-based groups are left skewed. Consistently, the first quartile of ratings is a 1, that is, at least a quarter of all reviewers (2986 across all groups, ie, 35.8% on average) writing a nonpositive review submitted the lowest possible rating. The medians displayed in the boxplots as orange vertical lines range from 2 to 3. Only 3 dominant topics were associated with a median rating of 3, namely “menstrual irregularities,” “weight gain,” and “loss of libido.” For all other dominant topics, half of all unfavorable reviews have a rating of ≤2.

The mean ratings per dominant topic are represented as green star. On average, reviews predominantly describing menstrual irregularities have the highest average rating (mean 2.92, SD 1.53; 1223/8330, 14.68%). The next highest average ratings were in reviews describing weight change (mean 2.87, SD 1.52; 807/8330, 9.69%) and loss of libido (mean 2.84, SD 1.49; 963/8330, 11.56%). Conversely, reviews with the lowest ratings, on average, predominantly described multiple cause dissatisfaction (mean 2.34, SD 1.45; 1493/8330, 17.92%), cramps and pain (mean 2.39, SD 1.55; 926/8330, 11.12%), and continuous bleeding (mean 2.45, SD 1.47; 965/8330, 11.58%). Skin problems (mean 2.53, SD 1.49; 1051/8330, 12.62%) and mental health problems (mean 2.57, SD 1.48; 902/8330, 10.83%) had slightly greater ratings than the bottom 3 groups.

## Discussion

### Principal Findings

Our findings are in line with the literature evaluating how users, mostly women, use social media to discuss and evaluate different contraception options. Side effects were the most important area that was discussed online. Our NLP algorithm extracted 8 topics, of which 7 clearly describe a specific unpleasant side effect and only 1 less concise topic also encompasses other issues such as general life challenges, relationship issues, or product warnings. The algorithm did not extract any frequent words indicative of nonhealth related challenges such as cost and accessibility.

Our research extends the existing body of knowledge in several aspects. First, we found that in the online drug review forum, niche products tend to be overrepresented compared to their prevalence among the respective populations in the United States. For example, Table 3 shows that 22.52% (4392/19,506) of the reviews discussed hormonal implants. However, their prevalence among women using reversible contraceptive products (either LARCs or short-acting methods) is 7.3% [2]. Similarly, vaginal rings (827/19,506, 4.24% of reviews vs 2.9% in the respective population [2]) and hormonal patches (800/19,506, 4.1% vs 1.1% [2]) appear to be overrepresented. Even more interestingly, IUDs are substantially underrepresented. While 18.82% (3671/19,506) of contraceptive product reviews discuss IUDs, they are used by almost a third of women [2] using female contraceptive products.

The underrepresentation of IUDs might be attributable to the fact that on average, women tend to be more satisfied with IUDs than with other contraceptive products (Figure 2; Table 3). In general, people are more likely to write an online review when they have a complaint than when they are satisfied [52]. Nevertheless, in Figure 2 and Table 3, we observe that a substantial share of women report positive experiences with contraceptive products, ranging from slightly >70% (2589/3671, 70.52%) of favorable reviews for IUDs to 46.9% (205/437) for reviews of POP. Overall satisfaction with reviewed birth control products may be even greater if the probable negative bias inherent in online reviews is accounted for.

Our NLP-based evaluation of product reviews also offered valuable insights into how women experience different product-based contraceptive methods and how negative experiences relate to the overall satisfaction with the method.

### Hormonal Short-Acting Contraceptive Methods

#### Overview

A first pattern we observed was that hormonal contraceptive methods with a higher level of systemic absorption, such as POP, birth control shots, and COCP, received greater shares of unfavorable reviews (232/437, 53.1%, 327/653, 50%, and 3968/8606, 46.1%, respectively) than methods with a lower level of systemic absorption, such as hormonal IUDs (848/2871, 29.54%). For copper IUDs, which do not release any hormones, only 29.3% (234/800) of reviews were unfavorable (Table 3). Thus, based on the online reviews and ratings, it appears that on average women in the website Drugs.com [43] sample are less satisfied with short-acting methods that rely on a systemic hormonal effect, which is in line with the findings of Merz et al [27] studying posts on X over time. This is particularly interesting as recent literature describes a trend of women turning away from hormonal contraceptives despite their high efficacy due to the influence of social media [26,36]. While clinical studies confirm a range of side effects with hormonal contraception, some researchers suspect they may be perceived to be more severe than they truly are [36]. However, our research suggests that women rate oral hormonal contraceptive products, hormonal implants, and shots less positively than nonhormonal methods and that this is linked with specific side effects.

#### Progestin-Only Methods and the Role of Irregular Bleeding Pattern

Figure 2 shows that POPs, contraceptive implants, and injectable contraceptives, which are all progestin-only methods, obtained comparably low average ratings with a high SD. According to the reviews, the most common side effects for these methods relate to irregular bleeding pattern and continuous bleeding (Figure 3). This is in line with the relevant women’s health literature describing irregular or unscheduled bleeding as their most common side effect (eg, [53]). The inhomogeneous experiences with these progestin-only methods might be—to some extent—explained by the interplay between users’ expectations and actual experiences. Although irregular bleeding resulting from these progestin-only methods decreases over time [54], women may be more disgruntled by the initial irregularity, especially if counseling focused more on the long-term than short-term expectations.

#### Potential Role of Ease of Administration for Cycle Control

Among the remaining short-acting contraceptive methods, hormonal patches and vaginal rings obtained comparably high average ratings and lower SDs than COCPs and extended-cycle OCPs (Figure 2). For COCPs, this is expected as women who are prescribed contraception for the first time often opt for the COCP due to its ease of initiation and discontinuation [55]. This initial usage likely leads to varied experiences.

However, this disparity in low average ratings of extended-cycle OCPs versus comparably high and more homogenous average ratings of vaginal rings and hormonal parches is unexpected, given the similarity of side effects across combined hormonal contraceptives (CHC) [53]. A potential explanation of our findings is that the patch and the ring may achieve better cycle control than the extended-cycle COCP due to the lack of need for daily administration [53]. Indeed, for extended-cycle OCPs, nearly half of the reviews (97/203, 47.8%) predominantly described abnormal bleeding, whereas this was only the case for 14% (22/157) of reviews of hormonal patches and 5.7% (17/297) of reviews of vaginal rings (Figure 3). Those opting for an extended-cycle OCP probably do so with the intent of significantly reducing or entirely ceasing their menstrual cycles, which is the primary distinction between standard and extended-cycle OCPs. Unfortunately, breakthrough bleeding is very common early in the use of an extended-cycle regimen [53]. Therefore, women who are hoping for no bleeding are likely to be unhappy with increased bleeding, especially if they are not warned about this. It is also possible that there could be other explanations, such as increased hormonal stability with nonoral administration of CHC [55].

#### Skin Issues

Research has consistently shown that CHC are beneficial for the treatment of acne [56]. It is surprising, therefore, that in our study, skin problems appear as a dominant topic for COCPs, patches, and 91-day cycle OCPs more commonly than for other methods. Further research would be helpful to explore these findings. One possibility is that the skin problems reported by reviewers are not only acne but also other problems, such as melasma, a hyperpigmentation disorder well known to be associated with oral contraceptive use [57]. However, an example investigation of reviews in which “skin problems” are discussed reveals that many reviews describe disappointment resulting from the birth control product not meetings their expectations with regards to acne control. For example,

I’ve been taking this birth control for about a week now, and I have already noticed some changes. My skin is also acne prone, and I was really hoping that this birth control would help with it. Without the pill, I usually have many bumps on my forehead, my chin is pretty red, and once in a while I will get cystic acne. Now that I’ve been taking it, I have many new pimples all over my face, like my cheeks and on my nose, where I have never gotten it before. It’s also given me MORE cystic acne which is a pain. I really wish that it could have helped, but before I switch off I want to wait a little longer to be sure.

This disappointment might make them more prone to leave a negative comment than women who experience other side effects.

#### Loss of Libido

Interestingly, the loss of libido is still associated with comparably high average product ratings. Unfavorable reviews predominantly describing a loss of libido ranked third in average rating (Figure 4). Our analysis also revealed that loss of libido is the most common dominant topic for vaginal rings (75/297, 25.3% of unfavorable reviews), almost double the proportion of any other method. This is interesting since controversial findings on this topic exist in the literature. It has been hypothesized that CHC may adversely affect sexual functioning by increasing sex hormone–binding globulin (SHBG), which then decreases available testosterone and leads to decreasing endogenous hormone production. Oral estrogens are known to increase SHBG via a first-pass hepatic effect [58]. It could be hypothesized that nonoral administration may have a smaller effect on available testosterone, although other research has shown that both oral and vaginal CHC increase SHBG and decrease free androgens [59]. It has also been hypothesized that administering CHC via a nonoral route, such as the use of a patch or ring, may mitigate effects on sexual function via increased hormonal stability. The ring could also exert a local estrogenic effect, improving lubrication [55].

Several studies have assessed the effect of the vaginal contraceptive ring on sexual functioning, with mixed findings [55,59], and a recent meta-analysis revealed a possible positive effect at 3 months but no effect at 6 months [60]. A larger cross-sectional nonrandomized analysis revealed that decreased libido was most common among users of shots, rings, and implants [8], which is more congruent with our analysis. It is also worth considering that direct hormonal effects are not the only way a method could affect libido; physical discomfort, vaginal dryness or irritation, and excessive bleeding are also expected to contribute. This is also illustrated in this example review:

I have been on NuvaRing for 5 months. Within a month I noticed a decrease in my sex drive, and I’ve had vaginal dryness which makes sex painful. Bad sex has effected other parts of my life.

Overall, based on the results of our study, it is plausible to anticipate that the systemic administration of hormones might lead to a greater incidence of side effects and lower satisfaction levels. This expectation is corroborated by our data, not only describing side effects with regards to irregular bleeding patterns, skin issues, and loss of libido, but also an increased frequency of complaints such as weight gain and mental health issues associated with these hormonal methods.

### Discussion of Insights on LARCs

In our exploratory study, we observe that, on average, women are highly satisfied with their IUDs. In fact, among all contraceptive methods, IUDs are given the highest average ratings on the drug review website (Table 3). This finding is corroborated by existing research indicating high satisfaction levels with this contraceptive method [56]. IUDs offer substantial advantages. The hormonal IUD is known for its ability to significantly reduce menstrual bleeding, with the 52 mg version being approved by the Food and Drug Administration in the United States for both contraception and heavy menstrual bleeding treatment. On the other hand, the copper IUD stands out as the sole nonhormonal choice that offers the convenience of not requiring action during each sexual encounter. Although both types of IUDs can cause undesirable bleeding-related side effects—typically breakthrough bleeding with the hormonal IUD and heavy periods with the copper IUD—these decrease over time [54,61]. We can reasonably assume that women opting for an IUD, which necessitates a medical procedure for insertion, would be well-informed and prepared for this.

However, our research indicates that for a limited group of women, IUDs appear to create major problems. Between 11.3% (90/800) and 12.37% (355/2871) of all written online reviews emphatically describe cramps and pain related to the insertion procedure or persisting pain. In fact, cramps and pain are the dominant topic in 41.9% (355/848) and 38.5% (90/234) of unfavorable reviews of copper and hormonal IUDs, respectively (Figure 3). We also see that, on average, the ratings of reviews where cramps and pain are the dominant topic are the second lowest, occurring only slightly above multiple cause dissatisfaction (Figure 4). This observation has substantial implications for IUD counseling practices. Although physicians typically inform women about the potential side effects of IUDs, our findings underscore the necessity for health care professionals to provide even more comprehensive counseling regarding the risk of temporary as well as lasting cramps and pain, which heavily hampers women’s well-being, and to offer pain control options for the insertion procedure.

### Limitations

Our study is subject to several limitations. First, the reviews and ratings in online forums may exhibit bias, often skewing toward negative experiences, and there may even be a risk of fake reviews. Consequently, our dataset may not accurately represent the broader population of women using birth control products. Nonetheless, this limitation does not detract from our study’s objective, which is to illuminate the experiences with different contraceptive methods women share on a drug review website. This study was intended to supplement traditional qualitative but informal information sources used by women and their partners. Consequently, our extensive analysis of nearly 20,000 online reviews arguably offers a more representative and robust overview than anecdotal evidence gathered from conversations with friends and family regarding birth control options. All the same, we note that the reliance on data from a single source (ie, the website Drugs.com) may introduce a bias. This could affect the findings. Although the drug review dataset dates from 2009 to 2017, reviewers might have already been influenced by social media influencers, who are increasingly expressing concerns about hormonal contraceptives, sometimes inflating the severity of side effects [36], and advocating for nonhormonal methods. Furthermore, the dataset only contains reviews of products. As such, natural contraceptive methods, such as calendar rhythm and withdrawal are not covered. Performing the NLP and sentiment analysis on other contraceptive product review websites could enhance the breadth and robustness of our findings but is outside the scope of this analysis.

Second, our categorization did not stratify by dose or regimen timing (eg, 21 vs 24 active pills) due to insufficient review information (eg, Loestrin could refer to multiple different products with the same active ingredients in different doses and durations), nor by progestin type to avoid small group sizes.

Third, there are important limitations inherent in the topic modeling of birth control product online reviews. While topic modeling offers valuable insights, it is crucial to acknowledge its constraints so that they can be addressed effectively in future research. One of the primary limitations is the subjectivity involved in choosing the right number of topics. In addition, topic modeling may not adequately capture rare or nuanced topics. In our study, which identified 8 topics, we observed 1 particularly ambiguous topic, “Multiple cause dissatisfaction.” This topic is frequently associated with vaginal rings and hormonal patches and occasionally encompasses other topics, potentially obscuring the clarity and precision of our overall results. Conversely, our algorithm effectively differentiates between “menstrual irregularities” and “continuous bleeding,” despite their similarities. Notably, women experience “continuous bleeding” as more problematic than “menstrual irregularities.” However, due to the overlap in these side effects and the associated words, some reviews scored highly for both topics.

Another limitation in topic modeling is the potential ambiguity in allocating reviews to specific topics, which stems from the inherent challenge of accurately capturing the thematic essence of the text. For example, a word such as “skin” in an unfavorable review does not necessarily imply a discussion about skin-related problems. Furthermore, one review may describe several topics or side effects (Multimedia Appendix 2). Thus, analyses that are based on reviews grouped by dominant topic may not fully reflect other potentially confounding aspects.

Despite these challenges, our analysis suggests that using TF-IDF and NMF for topic modeling with 8 topics is the most effective approach. In this setup, most topics, apart from “multiple cause dissatisfaction” and the occasionally intersecting “menstrual irregularities” and “continuous bleeding,” are well-defined by distinct symptom sets, differentiating them from others. The process involved careful consideration of both the interpretability and the distinctiveness of each topic.

Finally, given the exploratory nature of our study, we did not engage in statistical significance testing; consequently, we could not definitively determine whether the observed differences in contraceptive method ratings are systematic. This study was primarily descriptive and does not involve inferential statistical analysis or controlling for confounding variables. We also did not investigate potential interactions between different side effects, such as whether reports of mental health issues could lead to more negative evaluations of other symptoms. The suitability of the dataset for inferential statistics is questionable, as it does not meet several crucial assumptions for significance tests, such as normality, homoscedasticity within groups, or independence of observations.

In summary, our findings provide semiqualitative insights, highlighting the occurrence of certain side effects in the real world and how they are associated with online contraceptive product ratings. A deeper understanding of effect sizes, relationships, and causality requires further research.

### Conclusions

This study contributes to the understanding of how contraceptive methods impact women’s overall well-being, as interpreted from a large corpus of online user narratives. Our findings provide a complementary perspective to those derived from clinical trials or the adverse effects documented in pharmaceutical labels and package inserts. By leveraging NLP to analyze user reviews, we aimed to support women in choosing contraceptive options that are not only safe and effective but also reduce the likelihood of specific symptom clusters that could negatively affect their quality of life.

For instance, women seeking contraception may have specific concerns, such as potential effects on libido, skin health, or menstrual regularity. While no contraceptive option is completely free of side effects, it is crucial that women have access to information that enables them to make informed decisions about which side effects they are prepared to accept, guided by the experiences of others. Accordingly, our analysis empowers women to benefit from the collective insights and experiences of a large user base, supporting more informed decision-making. In addition, this information aids HCPs in offering personalized advice to women and their partners.

A key observation from our study is that all female contraceptive methods reviewed online are associated with a substantial percentage of negative ratings. Notably, no contraceptive method to be administered by women received <29% unfavorable evaluations. This finding underscores a significant opportunity for enhancement in the realm of female contraception. The objective for manufacturers would be to innovate and develop contraceptive methods that exert minimal or no negative impact on the well-being and quality of life. In light of this, there is a recent trend toward natural or calendar-based methods sometimes supported by digital cycle tracking tools. Depending on individual life circumstances and personal beliefs, these methods may constitute a viable alternative for some women despite the inherent risks resulting from use failure (the website Drugs.com [43] provides a comparison of efficacy and typical use failure rates). Greater awareness of the side effects of contraceptive products for women could guide couples in making joint decisions about contraception and a more equitable sharing of responsibilities by considering more options. A secondary insight from our study is that dissatisfaction was particularly common for contraceptive products that may result in irregular or continuous bleeding, especially when users may have expected reduced or absent menstrual bleeding. IUDs are generally rated more positively than other methods, although about 1 in 10 users report severe cramps and pain which are linked to very poor ratings.

In conclusion, a pivotal element of efficacious reproductive management is the provision of comprehensive information to women regarding the potential side effects of contraceptives and their likely impact on overall well-being and quality of life. Advancements in artificial intelligence in general and NLP in particular can help in extracting, aggregating, interpreting, and sharing this information. In a broader context, the empowerment of women to manage their reproductive health is acknowledged as a fundamental catalyst for economic advancement and the achievement of personal and professional aspirations, as emphasized by organizations, such as the United Nations, the World Health Organization, and the Organisation for Economic Cooperation and Development. Moreover, female sexuality transcends the dimensions of reproduction and birth control, encompassing aspects of pleasure and human connection, thereby enhancing overall well-being [62]. Access to suitable contraceptive methods and thorough information about these options are vital in facilitating this empowerment.

## Appendix

Multimedia Appendix 1 Examples of reviews by dominant topic.

Multimedia Appendix 2 Three example reviews and their topic values.

## Abbreviations

CHC: combined hormonal contraceptive
COCP: combined oral contraceptive pill
HCP: health care provider
IUD: intrauterine device
LARC: long-acting reversible contraceptive
NLP: natural language processing
NMF: nonnegative matrix factorization
OCP: oral contraceptive pill
POP: progestin-only pill
RQ: research question
SHBG: sex hormone–binding globulin
TF-IDF: term-frequency–inverse document frequency
UCI: University of California, Irvine
