# Supplementary material for: Insights on the Side Effects of Female Contraceptive Products From Online Drug Reviews: Natural Language Processing–Based Content Analysis
Source: JMIR AI. 2025 Apr 3;4:e68809. doi: 10.2196/68809 (PMC12006776; doi:10.2196/68809)
Supplement: Multimedia Appendix 1 [file ai_v4i1e68809_app1.docx]

Appendix 1. Examples of reviews by dominant topic

| **Topic** | **Example 1** | **Example 2** | **Example 3** |
| --- | --- | --- | --- |
| Weight gain | "I am currently on my fifth year on Nexplanon. When I first got it implanted. I noticed that I gained ALOT of weight. Within the first couple months I noticed my weight jump to 30lbs. I have struggled with my weight I questioned my doctor. Who said it doesn’t cause weight gain. Then when it was time to renew my implant I gained and additional thirty pounds!!! After that I told my doctor I was told to change my diet. I did lose 30lbs. By exercising and eating less but it was a struggle. I am stuck at the first thirty pounds I don’t have periods with this birth control at all! After talking with a friend of the family she did say that Nexplanon indeed caused weight gain. she had a similar experience. I’m not pregnant I guess that good. I don’t" | "Depo provera did a good job at protecting me against pregnancy. But I gained a lot of weight!! I didn’t gain much for the first 3 months I had it. Although, after about 6 months (2 shots) I noticed a significant amount of weight gain, and after almost a year I gained about 20-25 pounds. I was exercising, and changed my diet and nothing happened. The irregular bleeding &amp; other side effects were minor side effects to me. The weight gain was my major concern. After reading all of the other reviews for depo, I saw a lot of other women had issues with weight gain too, so I switched to nexplanon (implant), and for me it has had the same side effects as the shot BUT without weight gain. I’ve been able to lose about 10 pounds since I switched!!" | "Been on it for 3 months, 20 pound weight gain - always hungry and never full. No periods, but not worth the weight gain and uncontrollable appetite... Was managing weight very well prior to implant..." |
| Skin problems | "AWFUL. I had fairly clear skin before taking Sprintec and had gotten the implant taken out a couple months prior. But within less than a month I began to see my face break out on Sprintec. It eventually developed to the point where I got hormonal acne on my forehead and chin, places I had NEVER gotten acne. I stayed on the pill for 3 months and couldn’t take the acne any more, and the worst part is that the acne WORSENED after I got off. It has been four months and my face is filled with acne scars and new blemishes. This has been an ongoing battle for me, and I regret taking the pill. Immensely. Please avoid this at all costs! Especially if you have sensitive skin or acne free skin to begin with! I’d give anything to have my old face back." | "I’ve been taking this birth control for about a week now, and I have already noticed some changes. My skin is also acne prone, and I was really hoping that this birth control would help with it. Without the pill, I usually have many bumps on my forehead, my chin is pretty red, and once in a while I will get cystic acne. Now that I’ve been taking it, I have many new pimples all over my face, like my cheeks and on my nose, where I have never gotten it before. It’s also given me MORE cystic acne which is a pain. I really wish that it could have helped, but before I switch off I want to wait a little longer to be sure." | "Horrible, horrible, horrible I have never had acne this bad in my life!!!!!!!!!! My WHOLE chin and jawline are red and covered in cystic acne!!! I HAD PERFECTLY CLEAR SKIN BEFORE. I am honestly in a complete panic with what is going on with my skin. I’m in shock that a small pill could do this much damage. My face hurts so bad because of the acne. Its been only 3 weeks since I started taking it. Switching to sprintec tomorrow. DO NOT USE THIS, SAVE YOUR SKIN!!!!!" |

Appendix 1 (continued). Examples of reviews by dominant topic

| **Topic** | **Example 1** | **Example 2** | **Example 3** |
| --- | --- | --- | --- |
| Loss of libido | "I have been taking Ocella now for about 6 months and in those months I have experienced some weight loss as well as clearer skin. However I also have found that I have ABSOLUTELY NO sex drive. I am only 21 and before taking Ocella I had enough sex drive for me and other people, but now nothing." | "My sex drive completely disappeared and it caused me as well as my husband much distress and depression. Two weeks after stopping the medication, my sex drive came back with a vengeance. So happy I decided to stop taking this pill." | "I have been on NuvaRing for 5 months. Within a month I noticed a decrease in my sex drive, and I’ve had vaginal dryness which makes sex painful. Bad sex has effected other parts of my life." |
| Mental health problems | "I give this a two due to the simplicity of it. However, I am a completely different person. I have mood swings; I have daily or weekly mood swings, I’ll be ticked off for a whole day, then crying about nothing. I have had mood swings before and get over them. I don’t get over these ones. My boyfriend has told me I am not the same person. As everybody’s doctor has told them that everyone is different. I don’t have my period, which is okay but can’t handle the mood swings, I notice it during everyday life. I am actually getting it taken out." | "I used this pill during my teens and it caused irritability and heavy mood swings. Perhaps it was just teen angst but I tried microgynon recently, which uses the same hormones just different levels, and experienced similar mood swings and depression." | "I experienced mood swings and anxiety due to this prescription ... would not recommend." |
| Menstrual irregular-rities | "I have had nexplanon since February of 2015, it was great at first when I got it, no periods, no cramps, nothing. At the end of July I started spotting (which was normally when I would start a period before birth control). I just thought maybe it was just a few times a year I would have a small period. Well not even 2 weeks later I had a full blown period and heavy at that. Awful cramps and mood swings came with this period and it lasted longer than any period I had ever had before. After it was over another 2 weeks went by and I started my period again. This one is not that heavy but it’s really annoying me and I got so used to not having one I was spoiled. I am hoping that these multiple periods in a month stop." | "I was switched onto apri and I got my period for seven days when before I got it for only 3-5. I get severe cramps, bloating, and heavy periods. I will get spotting for about a week or two after I get my period. It has prevented pregnancy but does not help with any symptoms that come with periods." | "I have been on this medication for almost a month. I got my period once , but it hasn’t even been a week later that I got a second period. My first period was very light and only lasted three days, but I’m not sure how this period will be." |

Appendix 1 (continued). Examples of reviews by dominant topic

| **Topic** | **Example 1** | **Example 2** | **Example 3** |
| --- | --- | --- | --- |
| Cramps and pain | "My experience was absolutely horrible. Birth control works different for everyone but this was by far the worst pain I’ve ever been in. Immediately after inserted I was in excruciating pain. I started having heat waves and extreme cramping. This lasted for weeks. I had an ultrasound done to see if it was causing any ovarian cysts, or if the iud wasn’t inserted correctly and my ultrasounds came back normal. A week later I had to have it removed (after a month) because of how much pain I was in. I would highly recommend not having this at all, because of how severe my pain was. I had to take pain killers every 2 to 3 hours, and I would wake up in the middle of the night because of how much pain I was in. Absolutely terrible!" | "DON’T DO IT! I am 24, have a high pain tolerance, never have had any issue with my birth control, have never been pregnant, am healthy and active! The Skyla insertion was the worst pain I’ve ever had. I started balling crying. Once it was complete, I nearly passed out walking to my car. I was in severe pain all day and had to leave work. I figured all the pain would subside. I went home, used a heating pad, ate, took some pain killers but nothing helped. I had consistent spotting and cramping. I would usually work out every day... but I was in too much pain to do so. I waited about 10 days to have sex, only to bleed profusely and be in immense pain. I tried to wait it out, kept it in for about a month and eventually had to get it remove" | "I got the kyleena inserted today and experienced the worst cramps in my life. The insertion were (8/10) on the pain scale. I am not very sensitive to pain but can’t take any pain medication. The last 4 hours has been the worst in my entire life so far I have really bad cramps now 10/10 and nausea. I can’t even get out of bed because of the severe pain!" |
| Conti-nuous bleeding | "I got this implant on the 13th of November 2015... I’ve been bleeding since. Plus, acne ! Everywhere. I’ve been on accutane when I was younger and now I have acne all over again because of this implant. It is currently March and this marks my 6th month and I’m STILL bleeding. My gynecologist says it takes about 6 months for the bleeding to subside. If my bleeding doesn’t stop by the end of the month then I’m getting this out of me." | "I put my teenage daughter on Implanon 2 months ago and she has had almost non-stop bleeding ever since. The two times the bleeding slowed down to spotting she had horrible cramps after intercourse and started bleeding again." | "With liletta I have been bleeding for 3 month s I am so so tire of bleeding" |
| Multiple cause dissatis-faction | "Makes me feel very moody and sensitive, my husband and I fight all the time. When we got married I felt so much in love but know not sure about it. He said I changed a lot after having our baby. So not sure if the IUD is making me feel that way. I feel so bad because I get mad very easy for little things and I feel like I am loosing my husband. Of course that he doesn’t want to wear his ring makes me think things but he said that he is not use to wear rings and I always wear mine. I cook breakfast every single day, cook lunch for us to take it to work since we do not have to much money and sometimes I feel that he doesn’t really appreciate it! Do laundry, clean and he doesn’t really help me much and he doesn’t see it. Not sure what to think" | "I started the Nuvaring about two weeks ago. And for the first couple of days I felt nothing. I loved it and thought it was going to be a smooth experience. Well I was wrong. I soon begin to once in a while feel headaches and lots of tiredness. Then I did not feel myself at all. I felt like I had no feelings whatsoever to anyone at all. It was making me not even want to have sex at all so what was the point of even getting on it?? Even my mom and boyfriend said I didn’t seem myself. I was super moody and kind of depressed. I took the ring out last night and I already feel better and back to my happy self. Do not recommend" | “Do not take this pill.” |
