# Supplementary material for: Insights on the Side Effects of Female Contraceptive Products From Online Drug Reviews: Natural Language Processing–Based Content Analysis
Source: JMIR AI. 2025 Apr 3;4:e68809. doi: 10.2196/68809 (PMC12006776; doi:10.2196/68809)
Supplement: Multimedia Appendix 2 [file ai_v4i1e68809_app2.docx]

Appendix 2. Three exemplary reviews and their topic values

| Topic | ”EXTREME fatigue and weight gain, loss of period, sore breasts, nasuea. I started taking pill and couldn’t get enough sleep after 8- 10 hours of sleep I would need a morning nap for after dropping children at school - sometimes for 2 hours. Legs felt heavy. Gave it a try for 2.5 months and couldn’t stand it any longer. I’ve been off the pil for a week and feel completely normal again- thank goodness. I had an minena IUD with hormones for 5 years and believe that was giving me fatigue as well as it was slightly infected- but at that stage I had a series of life challenges that could have accounted fatigue too. It seems additional hormones do no suit me. Previous Oral birth control gave me severe depression. Still searching.” | ”DO NOT GET THIS IMPLANT! I was on my menstrual for about the usual time, almost a week, when I got my implant. The day getting it, I stopped menstruating for the day and fallowing day. Then to my surprise, I started again and it went on for two months straight. Not to mention the MOST PAINFUL cramps I have ever had in my life. When the horror stopped, I was free from pain and bleeding for only two weeks before the nightmare continued. Currently, I am scheduling my removal Immediately. And I am having those horrid cramps again. By the way, the removal is horrible.” | ”I miss my sex drive! I feel no sexual desire anymore since having the rod put in and I have gained exactly a stone in weight. I used to be one of those lucky girls who could eat what I like when I like, not anymore! Thanks to this weight gain I have horrible stretch marks on my legs and hips and feel the most unhappy I have ever felt. I’m 22 years old and have given the rod a chance for a year and I’m having it removed. ” |
| --- | --- | --- | --- |
| Weight gain | 0.026 | 0.000 | 0.053 (dominant topic) |
| Skin problems | 0.003 | 0.005 | 0.000 |
| Loss of libido | 0.003 | 0.000 | 0.048 |
| Mental health | 0.008 | 0.000 | 0.000 |
| Menstrual irreg. | 0.009 | 0.024 | 0.000 |
| Cramps and pain | 0.028 | 0.056 (dominant topic) | 0.000 |
| Continuous bleeding | 0.000 | 0.050 | 0.000 |
| Multiple cause | 0.037 (dominant topic) | 0.006 | 0.032 |
